# The Impact of Non-Nodulating Diazotrophic Bacteria in Agriculture: Understanding the Molecular Mechanisms That Benefit Crops

**DOI:** 10.3390/ijms231911301

**Published:** 2022-09-25

**Authors:** Flávia Thiebaut, Maria Clara de Oliveira Urquiaga, Aline Cardozo Rosman, Mirielson Loures da Silva, Adriana Silva Hemerly

**Affiliations:** Laboratório de Biologia Molecular de Plantas, Instituto de Bioquímica Médica, Centro de Ciências da Saúde, Universidade Federal do Rio de Janeiro, Cidade Universitária, Rio de Janeiro 21941-902, Brazil

**Keywords:** bioinoculants, non-legume plants, non-nodular bacteria

## Abstract

Agriculture is facing increasing challenges with regard to achieving sustainable growth in productivity without negatively impacting the environment. The use of bioinoculants is emerging as a sustainable solution for agriculture, especially bioinoculants based on diazotrophic bacteria. Brazil is at the forefront of studies intended to identify beneficial diazotrophic bacteria, as well as in the molecular characterization of this association on both the bacterial and plant sides. Here we highlight the main advances in molecular studies to understand the benefits brought to plants by diazotrophic bacteria. Different molecular pathways in plants are regulated both genetically and epigenetically, providing better plant performance. Among them, we discuss the involvement of genes related to nitrogen metabolism, cell wall formation, antioxidant metabolism, and regulation of phytohormones that can coordinate plant responses to environmental factors. Another important aspect in this regard is how the plant recognizes the microorganism as beneficial. A better understanding of plant–bacteria–environment interactions can assist in the future formulation of more efficient bioinoculants, which could in turn contribute to more sustainable agriculture practices.

## 1. Introduction

Nitrogen is considered an essential macronutrient and primordial element for plants; it is present in the constitution of the most important biomolecules, such as ATP, NADH, NADPH, chlorophyll, proteins, amino acids, and numerous enzymes [1,2]. However, biologically available nitrogen is often in short supply for plants, thus limiting plant growth and primary production [3]. In this way, modern agricultural practices are very dependent on this mineral to maximize crop production. Furthermore, fertilization with this nutrient has been essential for agricultural production in keeping pace with the growth of the human population [4,5]. Nonetheless, nitrogen emissions (such as ammonia, nitrogen oxide, and nitrous oxide) contribute to global climate change and can cause serious health problems [6]. Currently, 107 million tons of nitrate are used as fertilizer in agriculture worldwide [7]. The Asia-Pacific region is the largest consumer, followed by the USA and Brazil, which is fourth among all countries, using about 4.55 million tons of nitrate. It is worth noting that the four countries that consume the most nitrogen fertilizer are the ones that have the highest percentage of crop production globally. However, Brazil ranks third in crop production, ahead of the USA. Although nitrogen fertilizer is required for most crops, improving agricultural N management focuses on synchronizing N demand and supply across crops, as well as improving crop N use efficiency. Overall, the efficiency of nitrogen use by plants is low; it is believed that 67% of all applied N is unaccounted for, and is ultimately lost in the soil system or emitted into the atmosphere [8]. Faced with this scenario, the world needs new alternatives to minimize the negative effects of chemical fertilizers on the environment and to reduce agriculture production costs without changing productivity.

The use of beneficial microorganisms, such as diazotrophic bacteria, has emerged as a sustainable alternative for agriculture. These bacteria can convert available atmospheric N_2_ into ammonia through a process known as “Biological Nitrogen Fixation” (BNF); the nitrogen is then taken up by plants for their metabolic functioning [9]. According to the FAO index, it is estimated that in 2018 34 million tons of nitrogen were fixed in the world, with the two countries that contribute the most being the USA (29.62%) and Brazil (27.82%) [10]. However, the establishment of a beneficial plant–bacteria interaction depends on factors such as plant species and genotype, bacterial strains, and environmental factors [11,12,13]. Diazotrophic bacteria species are phylogenetically diverse, having the ability to develop different types of root associations with several plant species. In the best-studied association, symbiosis and development of root nodule structures occur, with the bacteria being an endosymbiont [14]. In the other type of associations, bacteria are usually classified as non-nodular and can live inside plant tissues (named endophytic), be associated with roots (named associative), or be free-living in the rhizosphere [15]. The nitrogen-fixing nodulating bacteria are mainly of rhizobia genera, with association restricted to leguminous plants; these form nodules in the root, where the BNF occurs [14]. The main studies of non-nodular diazotrophic bacteria have identified the association of non-legume plants with the genera *Azospirillum*, *Azorhizobium*, *Azoarcus*, *Bacillus*, *Burkholderia*, *Citrobacter*, *Enterobacter*, *Gluconacetobacter*, *Herbaspirillum*, *Klebsiella* and *Pseudomonas* [16]. Remarkably, all of these bacterial species are gram-negative, except for the genus *Bacillus*, which has gram-positive representatives such as *Bacillus subtilis*. These endophytic and associative diazotrophic bacteria are considered plant growth–promoting rhizobacteria (PGPR), as they improve plant performance by enhancing the availability of nutrients and improve soil fertility, mainly through BNF and phosphate solubilization [17,18,19]. In addition, they produce plant growth regulators and are involved in the modulation of phytohormone and defense responses [20,21,22], production of antioxidants, osmotic adjustment, and plant tolerance against biotic and abiotic stresses [23,24]. A large number of important crops in agriculture are non-nodulating grasses, such as maize, rice, wheat, sorghum, and sugarcane, showing the importance of studies and production of bioinoculant non-nodulating diazotrophic bacteria.

It is worth mentioning that the Brazilian group of researchers coordinated by Johanna Döbereiner was a pioneer in the identification of diazotrophic bacteria in non-legume plants [25]. Due to this study, in 1997, Johanna Döbereiner was nominated by the Brazilian Academy of Sciences for the Nobel Prize in Chemistry, bringing Brazilian research to worldwide recognition. In addition, since the 1950s Brazil has been gaining prominence with the advancement of knowledge about biological nitrogen fixation. Brazilian researchers have observed that interaction with diazotrophic bacteria allows sugarcane plantations to be cultivated with less use of nitrogen fertilizer with no loss in yield [26]. Thus, Brazil is a pioneer and international reference in studies on these bacteria and their use as bioinoculants [26]. The application of bioinoculants in agriculture can reduce chemical fertilizer use, as most of these are nitrogen-based, generating potential savings of billions of dollars per year for Brazilian agribusiness [27]. There are several types of bioinoculants containing living microorganisms that represent an eco-friendly alternative to the use of chemical fertilizers and pesticides in agriculture [28]. Used as biofertilizers and biocontrol agents, bioinoculants are predominantly based on bacteria and fungi that can promote plant growth and health. Bioinoculants with diazotrophic bacteria in their formulation can be used as both biocontrol and/or biofertilizers. Currently, there is an extensive list of diazotropes that are recommended as bioinoculants in Brazil; these can be applied both in legumes and in other crops, such as rice, wheat, maize, and eucalyptus [29]. About 85% of soybean plantations in Brazil rely on the application of bioinoculants [27], which are capable of fixing approximately 300 kg of N ha^-1^ with an efficiency of use close to 100% [30]. In addition, the N left in soybean crop residues can contribute to successive crops such as corn and wheat. The application of bioinoculants in the cultivation of grasses in Brazil is noteworthy as well [31]. Following advances in the use of bioinoculants, the search for patents on this topic has grown significantly. As of 2020, 76 patent applications had been made in Brazil for agricultural inoculant formulations, although only 16 were granted [32]. However, considering only patents using endophytic bacteria, an acceptance percentage of around 50% in the number of patents applied for can be observed.

**Figure 1:**
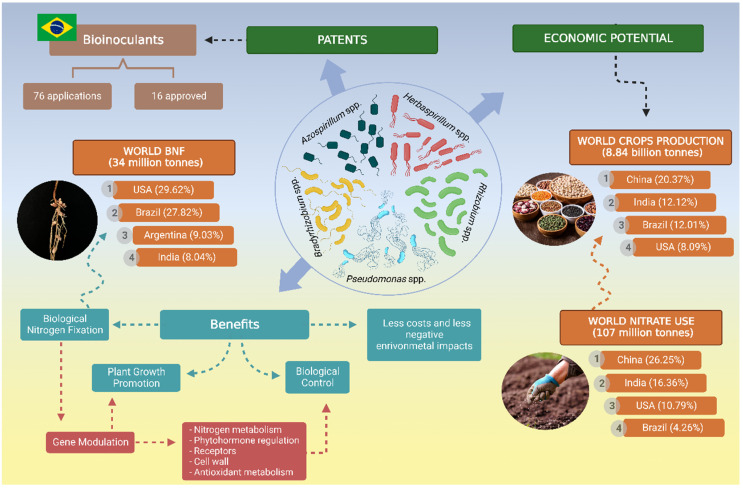
The impact of diazotrophic bacteria for more sustainable agriculture practices. Diazotrophic bacteria can bring great benefits to plants, including biological nitrogen fixation, plant growth promotion, tolerance to stresses, and biological control, as well as positive economic and environmental impacts. The mechanisms involve modulation of plant gene expression in key metabolic and physiological pathways. The use of bioinoculants can contribute to more sustainable agriculture, including economic potential, by reducing the costs associated with the use of nitrogen fertilizers. Brazil is one of the countries that is leading biotechnological research in the area of bioinoculants, represented by the growing number of patent applications for formulations of bioinoculants. Created with BioRender.com accessed on 31 August 2022.

Studies in this area have increased greatly, with the intent of developing more sustainable agricultural practices. Figure 1 summarizes the economic potential and the benefits for plants when using diazotrophic bacteria as bioinoculants. For this special issue, we carry out an overview of the research in the area, focusing on advances in knowledge about the molecular mechanisms that regulate plant interaction with non-nodulating diazotrophic bacteria and the benefits to agriculture from this association.

## 2. Plant Microbiomes and Prospection of Diazotrophic Bacteria

Plants are sessile organisms that have highly distinct microenvironments in their rhizosphere, surface tissues, and internal tissues, all of which harbor complex communities that include a wide diversity of microorganisms. These are known as plant microbiota, and the microbiota genomes that are closely associated with plants are collectively called the plant microbiome [33,34]. Recently, many significant steps have been taken towards understanding several aspects of the diversity and dynamics of plant microbiomes and the benefits provided by these microorganisms, which include diazotrophic bacteria [35,36,37,38].

Deeper knowledge of DNA composition and the development of molecular biology techniques, including next-generation sequencing methodologies, have established a revolution in bacterial identification. Currently, modern analysis of plant microbiomes integrates omics data for both the host and the microorganisms [39]. The emergence of metagenomics, based on the description of all DNA sequences in environmental samples [40], has revealed the genomic and taxonomic landscape of microbial communities that live in specific ecological niches in the rhizosphere [33,41]. These metagenomic sequencing analyses generate large amounts of data that require further analysis to obtain significant results [42]. Such analysis can elucidate the presence of functional redundancy or overlapping genomic characteristics in most PGPR, allowing the discovery of new genera and species [43].

One of the most widely recognized advances is the high-throughput sequencing of the smaller subunit of ribosomal RNA, the *16S rRNA* gene, which for the first time made it possible to establish a hierarchical taxonomic system based on an efficient molecular marker [44]. This amplicon corresponds to a highly conserved genomic region present in all bacterial cells which is essential for knowledge of the evolutionary relationships between the rhizospheric microbiota [17,45,46,47]. In parallel, the nuclear ribosomal internal transcribed spacer (ITS) region has been used as the main microbial marker gene for fungi [48].

In addition, several of the methodologies that contribute to bacterial genomic characterization are based on characteristic variations in genomic restriction sites, such as the occurrence of insertions and deletions, repetitions of DNA sequences or micro-satellites, single nucleotide polymorphisms, or other sequence variations distributed in bacterial genomes, which can determine a profile of DNA bands [49]. These banding patterns or DNA fingerprints are then used to compare isolates in order to assess the intraspecific diversity present in the plant microbiome [19,50]. Most DNA fingerprinting techniques are based on the presence or absence of restriction sites, while others are based on the homologies of short oligonucleotide primers [51]. Several DNA fingerprinting methodologies have been developed and improved since the 1970s, and many continue to be used today [49].

Many studies based on plant–microbe interactions have deepened our understanding of microbial interactions in the context of promoting benefits and discovering the factors that shape microbial diversity [18,19,52,53]. Studies have pointed out that the microbial population is generally higher in the rhizosphere than in the soil due to the secretion of root exudates that contain secondary metabolites, which are energy sources for microorganisms [54,55]. One line of studies of plant microbiomes is mainly based on the isolation, characterization, and prospection of new PGPR, followed by evaluation of the performance of these microorganisms in plant development to produce novel bioinoculants [17,50]. Interestingly, bioinoculants can be applied to soil and crop seeds either as a single inoculant or in combination as a microbial consortium, thereby maximizing their effect. Thus, the manipulation of microbiomes represents away to increase plant growth and productivity without the consequent environmental pollution associated with indiscriminate use of chemical fertilizers.

### Prospection of Diazotrophic Bacteria

Research focusing on the interaction between sugarcane and diazotrophic bacteria revealed that its microbial ecosystem is self-sustaining and capable of maintaining nitrogen fixation even under field conditions [36,56,57,58,59,60]. Among the diazotrophic bacterial genera, *Beijerinckia*, *Gluconacetobacter*, *Azospirillum*, and *Herbaspirillum* [61,62,63] are the most prominent and have been the focus of many isolation and cultivation approaches. A comprehensive picture of the structure of bacterial and fungal communities associated with sugarcane has been described, identifying 23,811 bacterial operational taxonomic units (OTUs) through amplicon sequencing of the *16S rRNA* gene [64]. In this study, the authors identified bacterial genera containing PGPR, including *Azospirillum*, *Bacillus*, *Beijerinckia*, *Bradyrhizobium*, *Erwinia*, *Enterobacter*, *Herbaspirillum*, and *Gluconoacetobacter*, which might potentially contribute to nutrient acquisition. A recent study focusing on evaluating *Bradyrhizobium* sp. community density in two commercial sugarcane cultivars confirmed the natural presence of diverse *Bradyrhizobium* spp. in these root systems [46]. This study combined DNA fingerprinting and *16S rRNA* sequence analysis, revealing high genetic variability. The sugarcane microbiome was exploited using the community-based culture collection (CBC) approach to select microbial groups based on community profiles targeting neglected microbial groups, thereby assembling a synthetic microbial community [65]. In the next step, maize was used as a model to probe the synthetic inoculant; as a result, inoculated plants were able to increase their biomass by 3.4 times compared to uninoculated plants [65].

Moreover, many investigations dedicated to studying N_2_-fixing microbiomes have focused on sequencing and identifying the *nifH* gene to isolate and characterize the diazotrophic community in sugarcane genotypes [36,59,60,66]. The *nifH* gene was amplified in nine of thirty samples from the four sugarcane species assayed, providing evidence for genetic variation in diazotrophic communities among different sugarcane species [36]. In parallel, *nifH* gene Illumina MiSeq sequencing revealed a significant difference in the diazotrophic communities of all five *Saccharum* cultivars sugarcane species, with most being located in the root [60].

Similar investigations have been performed in maize [50,52,67,68,69], rice [47,70,71], wheat [72], mung bean (*Vigna radiata*) [17], cowpea bean (*Vigna unguiculata* L. Walp) [53,73], juçara palm (*Euterpe edulis* Mart.) [18], and tomato (*Solanum lycopersicum*) [19]. Recently published studies focusing on legumes, such as mung bean and cowpea bean, have focused on accessing the bacterial community in the nodules as their main objective [17,53,73]. Overall, sequencing of the *16S rRNA* gene has shown a high diversity of plant-diazotrophic bacteria association, a finding that supports the development of new bioinoculants.

In addition to plant genotype, several studies suggest that there is a decisive refinement of the bacterial community in the rhizoplane in close contact with the plant host on the root surface and at the emergence points of the lateral roots [38,68,70,74]. Biofilm formation or specific adhesion mechanisms may be key factors in this enrichment step, essentially modifying the root microbiome structure. In a novel study, rice plants engineered with CRISPR/Cas9 to modify a flavone biosynthetic pathway generated apigenin-enriched rice plants that extruded apigenin into the rhizosphere [70]. Consequently, the increased production of this flavone stimulated biofilm formation in diazotrophic soil bacteria, improving the colonization of diazotrophic bacteria in rice plant tissues and promoting BNF [70]. Interestingly, a recent study carried out in nitrogen-depleted fields in Oaxaca, Mexico demonstrated that maize plants have developed an extensive network of mucilage-secreting aerial roots that harbor a microbiome rich in N_2_-fixing species [68]. The maize mucilage microbiome was explored through global genome sequencing and comparative bioinformatic analysis, showing that these genomes have high phylogenetic diversity.

**Figure 2:**
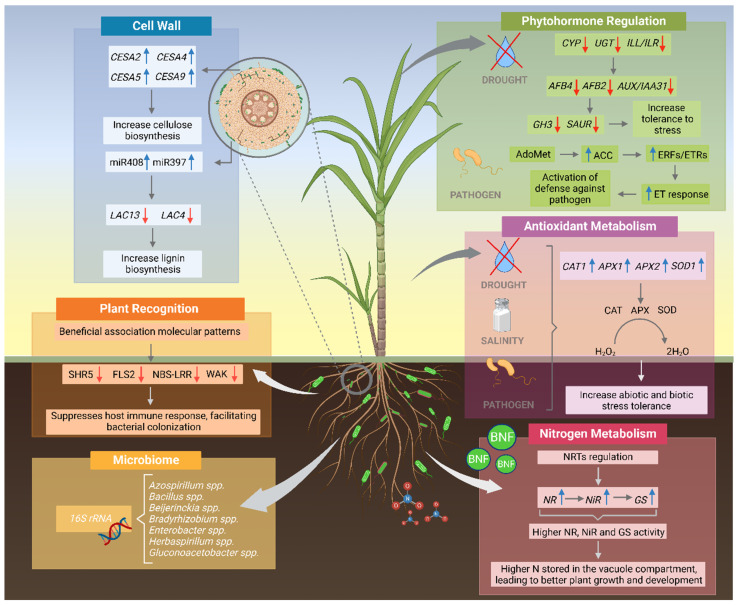
Molecular regulatory models identified in plant–diazotrophic bacteria association. The main studies involve the recognition via *16S rRNA* of the plant microbiome and the regulation of plant genes involved in nitrogen metabolism, phytohormone regulation, antioxidant metabolism, cell wall, and plant receptors involved in recognition of diazotrophic bacteria. Certain pathways lead to the promotion of growth and tolerance to biotic and abiotic stresses. Blue arrows: expression is up-regulated; Red arrows: expression is down-regulated; Gray arrow: flow of the pathway. ACC: 1-aminocyclopropane-1-carboxylic acid, AdoMet: adenosylmethionine, AFB2: Auxin signaling F-box 2, AFB4: Auxin Signaling F-Box 4, APX: ascorbate peroxidase, AUX/IAA31: Indole-3-Acetic Acid Inducible 31, CAT: Catalase, CES: cellulose synthase, CYP: cytochrome P450s, ERF: ethylene response elongation factor, ET: ethylene, ETR: Ethylene Receptors, FLS2: Flagellin-Sensing 2, GH3: Gretchen Hagen 3, GS: glutamine synthetase, ILL/ILR: IAA-Leucine Resistant (IRL)-Like, LAC: Laccase, N: nitrogen, NBS-LLR: Nucleotide-Binding Sites and Leucine-Rich Repeats, NR: nitrate reductase, NiR: nitrite reductase, rRNA: ribosomal RNA, SAUR: Small auxin-up RNA, UGT: UDP glucosyltransferases, WAK: Wall-Associated Kinases. Created with BioRender.com accessed on 31 August 2022.

It is worth noting that many bioinoculants on the market use a set of beneficial bacteria. Aiming at better efficiency in the use of diazotrophic bacteria in the formulation of bioinoculants, the identification of native plant microbiota is extremely important for both the bioprospecting of new strains and for a better understanding of the coexistence dynamics of the bacteria present in the bioinoculants and the crop microbial community. Thus, the 16S rRNA gene sequencing technique has been widely used to identify non-nodulating bacteria, as illustrated in Figure 2. However, the role of the rhizospheric microbiome in promoting plant growth is not yet fully understood.

## 3. Molecular Mechanisms Involved in the Association of Plant and Non-Nodulating Diazotrophic Bacteria

Many studies have already shown that diazotrophic bacteria can promote plant growth and resilience to various environmental stresses [17,18,23,24,75]. However, few studies have described the genetic and biochemical mechanisms involved in promoting benefits to plants during bacterial interaction. Cell biology and genomic approaches such as transcriptomics, proteomics, metabolomics, genetics, and epigenetics are making a great contribution to the understanding of the complex interactions that occur between plants and PGPR. Brazilian researchers have been pioneers in elucidating the regulatory networks involved in the promotion of plant-growth by non-nodulating diazotrophic bacteria as well as the mechanisms associated with increased tolerance to abiotic and biotic stresses. These important data are reviewed in the following topics.

### 3.1. Modulation of Nitrogen Metabolism

An important plant pathway that is differentially regulated by plant association with diazotrophic bacteria is nitrogen metabolism, which is involved in plant growth and development. Plants can obtain nitrogen from the soil in the form of nitrate and ammonium and via BNF through association with diazotrophic bacteria [16,76]. An interesting question to be addressed is whether the contribution of diazotrophic bacteria to better plant development involves nitrogen nutrition by directly providing ammonia, by improving plant N uptake from soil, by modulating the plant nitrogen metabolism genes, or through more than one of these routes.

Studies have shown that diazotrophic bacteria can upregulate important genes involved in nitrogen metabolism such as *NR*, *NiR*, and *GS*, which encode nitrate reductase, nitrite reductase, and glutamine synthetase, respectively, and collectively increase the activity of these enzymes in plants [11,21,76,77,78,79]. As illustrated in Figure 2, the modulation of nitrogen metabolism results in higher N storage in the vacuole compartment, leading to better plant growth and development. For instance, NR and NiR are the key regulatory enzymes of the nitrate assimilation pathway, reducing nitrogen absorbed as nitrate (NO_3_^−^) to nitrite (NO_2_^−^), which is in turn reduced to ammonium (NH_4_^+^), leading to plant N assimilation [80,81]. A recent study has shown that maize plants inoculated with *A. brasilense* sp245 or *H. seropedicae* HRC54 have higher expression of *NR* and *NiR* genes, respectively, when compared to non-inoculated plants [82]. Sugarcane plants inoculated with *Enterobacter roggenkampii* ED5, as well as wheat, maize, and cucumber plants inoculated with *Paenibacillus beijingensis* BJ-18, have shown higher expression of *NR* genes, indicating that these bacteria might stimulate nitrate reductase activity in these plants [76,83].

Ammonium is mainly assimilated with the glutamine synthetase (GS) and glutamate synthase (GOGAT) cycle or with the glutamate dehydrogenase (GDH) enzyme [84]. GS fixes ammonium on a glutamate (Glu) molecule to form glutamine (Gln). This Gln subsequently reacts with 2-oxoglutarate to form Glu, this step being catalyzed by the GOGAT. Previous studies have shown that two ESTs (expressed sequence tags) encoding a cytosolic form of GS1 are up-regulated by diazotrophic bacteria in wheat roots [85], while in sugarcane plants GS2 has been observed only in inoculated plants [86]. Wheat, maize, and cucumber plants inoculated with *P. beijingensis* BJ-18 show up-regulation of GS and GOGAT genes [76]. Furthermore, it has been observed that diazotrophic bacteria can increase the enzymatic activity of GS in wheat, maize, sugarcane, and cucumber and can increase the relative concentrations of glutamine, glutamate, and other amino acids [11,76,77,87,88].

Important genes that encode nitrate transporters have been identified as being differentially regulated in plants inoculated with diazotrophic bacteria. It is supposed that diazotrophic bacteria can stimulate plant growth through improved nitrogen uptake from soil, and as such this class of transporters is likely to play an essential role in plant–microbe interaction [89]. The genes encoding low and high-affinity nitrate transporter proteins NRT1.11 and NRT3.1 were transcriptionally induced in maize plants inoculated with *A. brasilense* sp245 and *H. seropedicae* HRC54, respectively [82], while NRT2.1 (low affinity) and NRT1.1 (dual affinity) were repressed in maize roots inoculated with *H. seropedicae* [90]. Wheat, maize, and cucumber plants inoculated with *P. beijingensis* BJ-18 and rice inoculated with *A. brasilense* or *H. seropedicae* showed up-regulation of several genes in the NRT family [76,89,91]. Thus, it has been proposed that the modulation of nitrogen metabolism genes by diazotrophic inoculation in plants can promote greater uptake of N from the soil, greater N assimilation, and/or increased storage of the reduced ammonium in the vacuole compartment, leading to better plant growth and development [11,82,90].

However, the success of this association may be dependent on the plant and bacteria genotypes as well as the N content present in the soil [76,79,83]. Several studies have shown that inoculation with diazotrophic bacteria may not influence nitrogen metabolism, although a greater accumulation of nutrients, growth, and development of plants can be observed [11,77,88,92]. These data suggest that the benefits of inoculating plants with diazotrophic bacteria could be mediated through different mechanisms, such as modulation of the plant nitrogen metabolism, the direct production of phytohormones, and/or the modulation of endogenous plant genes involved with phytohormones and defense, as discussed in the topics below.

### 3.2. Phytohormone Regulation

Considering that most of non-nodulating endophytic and associative diazotrophic bacteria produce plant hormones, another relevant question to be addressed is how the association of diazotrophic bacteria can modulate phytohormone production and consequently affect the entire performance of the plant. The phytohormones produced by diazotrophic bacteria can act as flexible signaling molecules, directly influencing plants’ gene expression, metabolism, and other physiological processes of plant growth and development [24,93,94]. In addition, bacterial colonization can modulate hormonal production by the host plant. Phytohormones can coordinate plant responses to environmental factors, leading to higher tolerance to biotic and abiotic stresses [24,94,95]. Figure 2 shows examples of phytohormone regulation leading to drought tolerance and pathogenic bacteria resistance stimulated by the diazotrophic plant–bacterial association.

Auxin is a major regulator of plant growth, development, and stress response [95,96]. Studies have shown that auxin is an important regulator of association between plant and diazotrophic bacteria [97,98]. Indole-3-acetic acid (IAA) is the main auxin in plants; certain endophytic bacteria can produce IAA and alter auxin levels [99]. Rice plants inoculated and then submitted to drought stress showed higher tolerance to stress, increased root growth and development, higher expression of the *IAA* gene, and higher concentration of IAA hormone [94]. Roots of rice inoculated with *H. seropedicae* showed that auxin-responsive genes were repressed, while maize inoculated with *A. brasilense* showed a significant increase in Auxin transporter-like protein 1 [93,100]. Remarkably, analysis of sugarcane genotypes with contrasting BNF efficiencies showed differences in auxin biosynthesis, auxin transport, and auxin signaling, as the auxin pathway was activated more in roots of the genotype that associated best with diazotrophic bacteria (i.e., high BNF) [79]. For instance, higher mRNA levels of transcription factor NAC1 were observed in a high-BNF sugarcane genotype. Furthermore, in maize inoculated with *H. seropedicae*, miR164, which regulates NAC1 levels, was repressed in comparison with non-inoculated plants [101]. As NAC1 is induced by auxin, leading to promotion of lateral root development [102,103], these data suggest that NAC1-miR164 might participate in a mechanism by which diazotrophic bacteria promote root development.

In addition, RNA-seq analysis of sugarcane plants inoculated with *G. diazotrophicus* and submitted to drought showed that all DEGs (differential expressed genes) annotated in roots as members of the auxin pathway were repressed, including genes for auxin biosynthesis/homeostasis (*CYP*, *UGT*, and *ILL/ILR*), signaling (*AFB4*, *AFB2*, and *AUX/IAA31*), and response (*GH3* and *SAUR*) [24]. This result suggests that sugarcane plants inoculated with *G. diazotrophicus* modulate these genes to help the plant tolerate water stress, again showing the benefits of inoculation. All of these studies together suggest that diazotrophic bacteria can modulate the auxin pathway both genetically and epigenetically, leading to promotion of plant growth, especially root growth, which depends on the association of the plant genotype with these bacteria.

Gibberellins (GA), which play an important role in plant growth promotion, can be produced by diazotrophic bacteria [104]. These hormones are naturally present in plants and regulate seed germination, root growth, root hair abundance, stem elongation, and leaf expansion [105,106]. The application of gibberellins has been reported to improve rice performance under saline stress as well as to reduce heavy metal stress [107,108]. Rice plants inoculated with *G. diazotrophicus* and submitted to drought showed higher tolerance to stress, increased root growth and development, higher expression of the GA gene, and higher concentration of GA_1_ and GA_3_ hormone [94]. RNA-seq analysis of maize inoculated with *A. brasilense* sp245 or *H. seropedicae* HRC54 showed that DEGs involved in GA signal transduction and response were induced in plants inoculated with both bacteria [82]. In other studies, it has been observed that genes involved in gibberellin synthesis were induced in maize plants inoculated with *A. brasilense* or *H. seropedicae* [100,109]. These data suggest that GA might contribute to the regulation of developmental adaptations in plants in response to beneficial bacteria stimulus.

Ethylene (ET) is another important phytohormone associated with plant–bacteria interaction. In plants, 1-aminocyclopropane-1-carboxylic acid (ACC) oxidase is essential in the ethylene biosynthetic pathway. Many diazotrophic bacteria are able to degrade ACC through the enzyme ACC deaminase and then use the degradation products as a nitrogen source [110,111]. For instance, *H. seropedicae* encodes ACC deaminase, which likely modulates ethylene production [93,112]. At optimal levels, ethylene is involved in plant development and natural tissue senescence and abscission; however, when overproduced it can decrease plant performance [113,114]. A dual RNA-seq analysis of wheat roots colonized by *A. brasilense* showed a decrease in the expression of *ACO*, which encodes for ACC oxidase that then catalyzes the conversion of ACC to ethylene, suggesting a decreased amount of ethylene production in inoculated wheat roots [85]. In another study, it was observed that ACC oxidase mRNA levels were downregulated; ethylene production was reduced approximately three-fold in rice roots colonized by *H. seropedicae* [115]. Similarly, in rice–*A. brasilense* interactions ethylene synthesis was repressed [89]. Multiple studies have suggested that the repression of ethylene is necessary to allow for plant–diazotrophic bacteria association [85,89,91]. In addition, decreased ethylene levels allow plants to be more resistant to a wide variety of environmental stresses [99], suggesting a great positive influence on inoculation with diazotrophic bacteria. *A. brasilense* colonization in wheat might suppress the inhibition of root cell elongation promoted by ethylene as reflected in the improvement of root systems of colonized plants [85]. The ethylene biosynthesis pathway was repressed in rice roots associated with *A. brasilense* and *H. seropedicae* [91]. In addition, ethylene responses in rice are controlled by both plant and bacterial genotypes, suggesting that this genetic combination might be involved in determining successful plant–bacteria association [116]. In the EST database, certain genes in the ethylene response pathway were differentially expressed in response to inoculation with *G. diazotrophicus* or *H. rubrisubalbicans* [117,118]. Sugarcane showed strong and specific induction of the expression of an ethylene receptor (ScER1) that is a negative regulator of the signaling pathway [119]. In contrast, a drastic repression of ScER1was observed in a pathogenic interaction, suggesting that ScER1 might discriminate between beneficial and pathogenic bacteria in the activation (or not) of defense responses [119]. This observation leads to another important question: how do plants orchestrate the hormonal responses that activate their defenses against pathogens and discriminate them from beneficial bacteria?

Biotic stresses are capable of causing severe damage to food production, leading to considerable pre- and post-harvest losses. In this context, many PGPRs trigger important mechanisms in biotic stress resistance, aiding in plant bioprotection [120,121,122,123]. The bacterial species *B. subtilis* exhibits direct and indirect biocontrol mechanisms to suppress disease and provide resistance to pathogen-caused pests, including signaling by phytohormones such as ethylene [120,122,123,124,125] (Figure 2). ET induces the transcription of genes encoding other pathogenesis-related proteins (PR), such as cellulase, chitinases, peroxidase, and chalcone synthase [126,127]. Bacteria such as *Acetobacter* sp., *Azobacter* sp., *Azospirillum* sp., *Pseudomonas* sp., and *Bacillus* sp., in addition to promoting plant growth, act as induced systemic resistance (ISR) voters in inoculated plants [120,128]. ISR activation by *B. subtilis* induced jasmonate (JA) synthesis, ET, and NPR1 (Non-Expressor of Pathogenesis-related genes 1) gene regulation in plants [120,129]. Cotton seeds (*Gossypium hirsutum* cv Deltapine Acala 90) treated with *B. subtilis* UFLA285 promoted resistance against damping-off disease, caused by *Rhizoctonia solani*, via JA/ET signaling [130]. Similar results were observed previously in *Arabidopsis* inoculated with *B. subtilis* FB17, which showed overexpression of PDF1.2 in the simultaneous presence of *B. amyloliquefaciens* FZB42 [131] and the pathogen *R. solani*, suggesting synergistic activation of the JA/ET pathway [132]. Thus, pathogen restriction and disease progression in plants can occur as a function of inoculation with PGPRs that activate the SA/ET-dependent ISR and NPR1 mechanisms [133]. Another example is the production of ACC deaminase by *Pseudomonas migulae* 8R6 in inoculated grape plants, which assists plants in regulating the levels of the stress-related hormone ethylene against Flavescence dorée phytoplasma, a disease that causes damage to grape crops [120,134]. In general, the biological impact of PGPR inoculation occurs via ISR induction mediated by signaling molecules such as jasmonic acid and ethylene.

Similar to ethylene, abscisic acid (ABA) is a hormone produced by plants in response to different types of stress [135,136,137]. RNA-seq analysis of sugarcane plants inoculated with *G. diazotrophicus* and submitted to drought revealed repression of ABA biosynthesis in roots, and the inoculated plants were more drought tolerant than non-inoculated plants [24]. In maize, RNA-seq analysis showed repression of all DEGs involved in ABA biosynthetic process in the inoculated plants [82]. Thus, diazotrophic bacteria have been reported to increase the tolerance of plants to abiotic stress, thereby decreasing stress-related ABA accumulation.

All of these results together show that endophytic and associative diazotrophic bacteria modulate diverse phytohormone pathways, with the efficiency of association resulting in differences in phytohormone regulation. In addition, due to the vital importance of phytohormones in plant metabolism and development, the manipulation of this pathway contributes to plant adaptation to different environmental stimulus, including resistance against certain pathogens, thus classifying this application as biological control.

### 3.3. Antioxidant Metabolism

One of the most widely studied response mechanisms to biotic and abiotic stimulus is the reduction in the accumulation of reactive oxygen species (ROS) in plant tissues [138,139]. An increase in ROS accumulation results in severe loss of crop productivity, affecting several cellular functions by damaging nucleic acids and oxidizing proteins [140]. Studies have revealed that diazotrophic bacteria can modulate the activity of antioxidant enzymes that detoxify ROS, mainly in plant leaves, conferring resistance to oxidative and abiotic stresses [20,22,23,141,142,143,144].

Different studies have tried to understand how the negative effects of oxidative stress are mitigated by modulation of antioxidant metabolism in plants inoculated with diazotrophic bacteria. Foliar spraying of maize with *A. brasilense* led to up-regulation of genes related to oxidative stress in leaves, such as *APX1*, *APX2*, *CAT1*, *SOD2*, and *SOD4* [22]. However, the highest expression was observed when foliar spraying was combined with the application of bacterial metabolites [141]. In soybean, the application of biological inoculants containing *Azospirillum* or their metabolites promoted plant growth and induced tolerance to oxidative stresses [142]. In three sugarcane cultivars inoculated with a consortium of five N_2_-fixing strains, high levels of ROS were neutralized by an increase in the activity of the antioxidant enzymes SOD and APX in young plants [145].

A correlation between modulation of antioxidant metabolism and tolerance to stresses in inoculated plants have been investigated. A beneficial bacterial consortium containing *Bradyrhizobium* spp., *A. brasilense* strains, and microbial secondary metabolites was found to mitigate oxidative damage in soybean cultivated under moderate drought by reducing leaf hydrogen peroxide content, proline and lipid peroxidation, and the enzyme activities of SOD, CAT, and APX [20]. On the other hand, red rice plants inoculated with *G. diazotrophicus* PAL5 and subjected to severe drought stress revealed a significant increase in SOD, CAT, and APX activity as compared to non-inoculated plants, which was due to positive regulation of the expression of superoxide dismutase (*sodA*), glutathione reductase (*gor*), and catalase (*katE*) [23]. Similarly, the presence of *A. brasilense* stimulated more activity by antioxidant enzymes in tree species such as *Cecropia pachystachya*, *Cariniana estrellensis*, and soybean, triggering increased drought stress tolerance [146,147]. Under salt stress, maize plants inoculated with *A. brasilense* Ab-V6, *R. tropici*, and co-inoculation with both bacteria showed up-regulation in leaves of genes related to antioxidant activity, such as *APX1*, *CAT1*, *SOD2*, and *SOD4* [141]. Furthermore, *Glycyrrhiza uralensis* plants associated with *Bacillus* sp. G2 under saline stress showed a drastic decrease in superoxide and hydrogen peroxide radical content through an increase in the activity of the antioxidant metabolism composed by the enzymes SOD, POD, CAT, and APX [148]. Taken together, these studies suggest that certain diazotrophic bacteria species can alleviate the negative oxidative effects of drought and saline stress, and may represent an efficient biotechnological tool to increase plant growth under saline stress (Figure 2).

Furthermore, diazotrophic bacteria can act as biological controls via modulation of oxidative metabolism (Figure 2). For instance, inoculation of grape plants with *B. subtilis* (PTA-271) induced an oxidative burst, promoted the accumulation of phytoalexin metabolites, and regulated defense-related gene expression in both shoots and roots, including transcription factors (ACCsyn, GST, CHS, CHI) and PR proteins (PR1, PR2, PR3, PR5, and PR6) [120,149]. Similar results have been observed in rice plants inoculated with *Bacillus* sp. (L81) and *Aeromonas* sp. (AMG272); both strains promoted 90% protection against the pathogen *Xanthomonas campestris* by modulating APX and glutathione reductase (GR) and by inducing the activity of chitinases and ß-1,3-Glucanases [150]. *Aeromonas* sp. (AMG272) is a dizotrophic bacteria isolated from the rhizosphere of rice plants. In tomato, inoculation with *Streptomyces* isolates (strains IC10 and Y28) triggered defense against *Fusarium oxysporum* f. sp. lycopersici race 3 (FOL), the causal agent of Tomato Fusarium Wilt (FWT) through the induction of antioxidant enzymes involved in plant resistance against pathogens [151].

### 3.4. Modulation of Cell Wall Composition

The plant cell wall is highly dynamic, being remodeled during growth and development while contributing as the main mechanism for perception of biotic and abiotic stresses [152,153,154]. Moreover, the cell wall determines the size and shape of cells, regulates cell volume and turgor-driven expansion, and plays an important role in the functional specialization of tissues and organs [155]. Environmental stimuli can alter cell wall structural components, leading to an increase in the composition of receptors, proteins, carbohydrates, and lignin, which can activate signaling to maintain cell wall integrity and the plant defense system [154,156]. Therefore, various processes participating in the establishment of plant–diazotrophic bacteria association might involve regulation of cell wall pathways.

The association with endophytic diazotrophic bacteria begins with the attraction of bacteria to the host roots, followed by the bacteria attachment on the root surfaces, and finally the colonization of the emergence points of the lateral roots [157,158,159,160,161]. Previous studies have reported that a wide diversity of diazotrophic bacteria, such as *H. seropedicae* Z67, *H. rubrisubalbicans*, and *A. brasilense*, produce cell wall-degrading enzymes, including cellulases or pectinases [157,162]. These studies suggest that proteins of diazotrophic endophytic bacteria can directly lead to the modification of the plant cell wall to facilitate bacterial association and colonization of internal tissues.

In addition, bacterial association could modulate plant gene expression related to cell wall formation. Notably, efforts are being made to assess the role of cell wall formation pathways in interactions between grasses and endophytic diazotrophic bacteria [82,156]. Modifications in plant cell wall architecture and composition could have a role in plant growth, as bacterial association can lead to an increase in plant biomass, promoting plant development and changes in root architecture [11,163,164,165]. Inoculation with *H. seropedicae* SmR1 induced the expression of a gene encoding a β-D-xylosidase and repressed a gene encoding a polygalacturonase, suggesting cell wall remodeling in rice roots [166]. Additionally, the same study demonstrated a positive regulation of genes that encode for proteins similar to expansin11. In addition to promoting the elongation [167,168] and initiation [169] of root hairs, expansion promotes the loosening of plant cell walls, which has an effect on cell enlargement and various developmental processes in which cell wall modification occurs. Likewise, inoculation of *Arabidopsis* with the endophyte *B. phytofirmans* PsJN led to increased plant growth and changes in the cell wall [170].

Remarkably, studies indicate that the modulation of certain cell wall metabolism pathways can be differentially regulated by the diazotrophic bacteria genotype and/or plant genotype. In maize, RNA-seq analysis showed that *H. seropedicae* HRC54 induced most of the identified DEGs involved in cell cycle progression and cell wall formation, suggesting that this endophytic bacterium activated cell divisions and DNA replication [82]. In contrast, genes encoding callose synthase enzymes were only induced in plants inoculated with *A. brasilense*, showing an opposite expression profile during association with *H. seropedicae* [82]. Remarkably, cellulose synthase (CESA) genes such as *CESA2*, *CESA5*, *CESA9*, and *CESA4* were more expressed in roots of sugarcane genotype in which BNF is more efficient, suggesting that these bacteria can modulate cellulose biosynthesis [156] (Figure 2). Furthermore, the same expression profile in two BNF-contrasting genotypes has been observed in sugarcane plants inoculated with diazotrophic bacteria *G. diazotrophicus* PAL5 [156].

In addition to genetic mechanisms, diazotrophic bacteria can trigger specific epigenetic modifications that regulate development [171] and cell wall lignification [101,172]. Epigenetic studies on sugarcane plants have revealed the differential expression of miR408 in response to either diazotrophic bacteria, *G. diazotrophicus* PAL5, or the pathogenic bacteria *Acidovorax avenae* [172]. MiR408 was induced in response to beneficial association and repressed upon pathogenic infection, regulating the expression of mRNA encoding laccase, a multicopper enzyme involved in lignin biosynthesis [172]. The same expression pattern was found in maize plants inoculated with *H. seropedicae* [101]. Additionally, an increase in the expression of miR397, which targets laccase genes, has been reported in maize during association with *H. seropedicae* [101]. Interestingly, a higher increase in lignin biosynthesis was observed in Chunee sugarcane roots after inoculation with *G. diazotrophicus* as compared to SP70-1143 roots [156], indicating that endophytic diazotrophic bacteria does not activate the early defense response against bacterial colonization in grasses, thereby decreasing plant lignin synthesis [101,172]. Thus, the repression of laccase triggers a repression of lignin biosynthesis, and consequently facilitates inoculation of beneficial bacteria (Figure 2).

### 3.5. Modulation of Plant–Microorganism Recognition Pathways

In the association between plants and beneficial diazotrophic bacteria, the plants need mechanisms to recognize and distinguish the signals of beneficial bacteria from pathogens in order to induce appropriate responses to each situation. During the establishment of plant–bacteria interaction, endophytic diazotrophic bacteria explore tissues within the root and colonize intercellular spaces and xylem vessels [157,173]. The entry of endophytes into plant tissues involves the activation of the plant defense system [15,156,174,175,176]. However, there is a paradox, because while the plant normally defends itself against microorganisms, it needs to identify beneficial bacteria and allow them to associate with and eventually colonize its tissues. Overall, most described mechanisms dedicated to plant defense are focused on responses to pathogenic organisms, whereas mechanisms associated with beneficial endophytes avoid plant defenses to achieve harmonious interaction [175]. Recent evidence suggests that beneficial bacteria alter plant defense responses during colonization, reinforcing the idea that fine-tuning of receptors in the plant’s defense mechanisms, which is a poorly understood topic, separates benefit-associated molecular patterns from damage-associated molecular patterns (DAMPs) [177]. Depending on the stimulus, these pattern recognition receptors (PRRs) are activated or not, and the host plant responds to facilitate an endophytic symbiotic relationship or to prevent colonization of invading pathogenic microorganisms [178].

The process of recognition and colonization by diazotrophic bacteria includes several key regulators, such as plant genes and receptors [166]. The recognition and transduction of extracellular signals into the cells are essentially mediated by plant receptors that belong to the family of Receptor-like Kinases (RLK), including Leucine Rich Repeat containing Receptor-like Kinases (LRR-RLKs), Lectin Receptor-like Kinases (LecRLKs), Lys-motif receptors (LysM), and Wall-associated Kinases (WAK) [15,179]. RLK and Receptor-like proteins play crucial roles in plant immunity [180]. The main receptors involved in the recognition of bacteria are highlighted in Figure 2. Considering that the success of plant–bacterial interaction is largely governed by these membrane-located immune receptors, a central question is whether the diazotrophic bacteria in plants specifically trigger expression patterns of some of these plant receptors [82,164,166,173,181]. A leucine-rich repeat-containing receptor-like kinase (SHR5) was the first plant receptor identified as responsive to association with non-nodulating diazotrophic bacteria. In sugarcane, SHR5 expression was significantly down-regulated by inoculation with the diazotrophic bacteria *G. diazotrophicus*, *H. seropedicae*, *H. rubrisubalbicans*, and *A. brasilense*, while inoculation with pathogenic bacteria (*Agrobacterium tumefaciens* A281 and *Leifsonia* xyli subsp. xyli), pathogenic virus (*Mosaic virus*), and pathogenic fungus (*Puccinia melanocephala*) showed no significant difference in SHR5 mRNA expression [181]. In rice, lectin-like receptor kinase 7 and SHR5 were significantly repressed in the presence of *H. seropedicae* [106]. RNA-seq analysis of maize plants inoculated with two diazotrophic bacteria revealed that the vast majority of signaling receptors classified as RLK were induced in plants inoculated with *A. brasilense*, while many members of this receptor family, including SHR5, were repressed by *H. seropedicae* [166]. In addition, certain DEGs were simultaneously repressed in both diazotrophic datasets, such as *b120* with S-locus lectin domain of bacterial PRRs, *wak5* with epidermal growth factor (EGF) repeats, and the cytoplasmic receptor *rpp13* [166]. Corroborating this evidence, inoculation of rice plants with *A. brasilense* led to the induction of *LYK8* and AGC kinase expression. Furthermore, SHR5 was differentially expressed at both time points studied [89]. These results indicate that the ability of diazotrophic bacteria to suppress the host’s immune response may be a determinant of whether they are able to colonize and benefit plants.

A second interesting issue to be addressed is whether these differences in BNF efficiency may be a consequence of the efficiency of bacterial colonization within plant tissues, which could have a direct relationship with the regulation of expression of specific genes for recognition and/or defense of the plant. A recent study has demonstrated that the regulation of plant receptors known to be involved in plant–bacteria diazotrophic recognition, including NBS-LRR, FLS2, WAK, and SHR5, are more expressed in low-BNF sugarcane genotype (Chunee) than in the high-BNF genotype (SP70-1143) inoculated with *G. diazotrophicus* PAL5 [79]. This evidence suggests that such regulation may be crucial for efficient recognition of diazotrophic bacteria and the establishment of a beneficial association.

## 4. Conclusions and Future Perspective

An important sustainable agricultural strategy to maintain productivity and reduce the application of chemical fertilizers, especially nitrogen fertilizers, is to explore the use of beneficial microorganisms in agricultural systems. In this context, modulation of plant microbiota and application of bioinoculants containing diazotrophic bacteria are the major approaches being studied.

**Figure 3:**
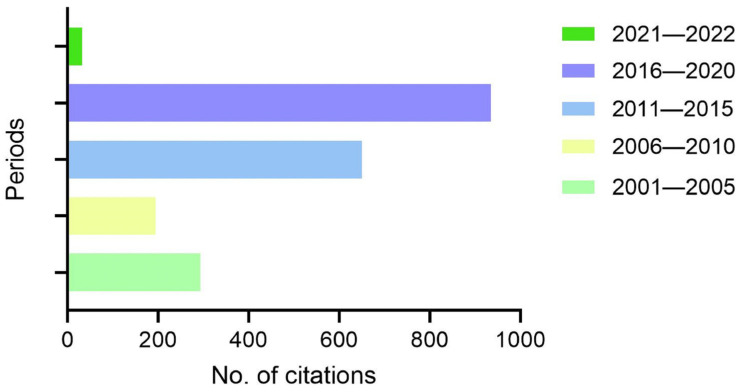
Absolute number of citations of Brazilian scientific publications involving use of molecular biology to understand the mechanisms triggered by plant–diazotrophic bacteria interaction in the period 2001–2022 (Source: Web of science—www.webofscience.com/wos/author/search, accessed on 28 August 2022).

Brazil is at the forefront in studies to identify beneficial diazotrophic bacteria as well as in the molecular characterization of this association on both the bacterial and plant sides. In particular, Brazil is a pioneers in the studies leading to application of diazotrophic bacteria as bioinoculants, as a large number of microorganisms naturally present in Brazilian soils have been isolated and identified for prospective use in agricultural systems [50]. Studies of Brazilian groups have been the basis for many other groups, as represented in Figure 3 by the number of citations of scientific publications involving molecular data on the plant–diazotrophic bacteria association.

The use of bioinoculants as replacements for chemical fertilizers is critical to sustainability. However, there is a concern that new agriculture practices ensure our environment remains both productive and healthy. Issues of efficacy and biosafety must be addressed, as bacterial species potentially harmful to mammals, including humans, have been isolated from plant rhizospheres [182]. A panel of tests, as well as an evaluation system, the Environmental and Human Safety Index (EHSI), have been proposed to assess the biosafety of bacterial strains used as bioinoculants [183]. It is worth noting that many of PGPRs, such as *Azospirillum* and *Azotobacter*, have already been classified as non-pathogenic (Risk Group 1/BSL1) [184].

Advances in the molecular studies in the area of plant–diazotrophic bacteria association have revealed that different molecular pathways in plants are regulated both genetically and epigenetically, providing better plant performance. In addition to modulation of the plant nitrogen metabolism, highlighted in this review, diazotrophic bacteria can contribute to the solubilization of phosphate [184]. Therefore, the application of bioinoculants could replace other fertilizers commonly used in agriculture as well. The promotion of plant growth by bioinoculants might be mediated through modulation of nutrient acquisition or phytohormone production. In this scenario, diazotrophic bacteria could help plants to become better adapted to the environmental changes, promoting greater tolerance to different abiotic and biotic stresses. However, there are gaps not yet filled in our understanding of plant–bacterial interaction and in ways to improve the efficiency of bioinoculants. A better understanding of gene regulation “in real time” in both plants and associated diazotrophic bacteria represents a powerful molecular tool to identify key genes and/pathways that could be manipulated, which could in turn optimize the use of bioinoculants. It is important to decipher the molecular and metabolic players involved in plant recognition of bacteria as beneficial microorganisms and distinguishing them from pathogens. Another aspect is how the environment contributes to regulating of the efficiency of a beneficial association. In addition, a broader study of the competition between beneficial microorganisms should be explored in order to improve bioinoculant formulations with different bacterial species.

In closing, the optimization of the use of bioinoculants in agriculture needs to translate the knowledge generated in the laboratory to application in the field. It is necessary to understand this intimate biological interaction from both sides, both plants and bacteria, as well as how it is affected by climate, soil, plant cultivars, and crop management. In addition, broad scientific dissemination is needed to spread this practice and make agriculture more sustainable.
